# Modeling data-driven clusters of social determinants of health (SDoH) and their associations with US suicide from 2009-2019

**DOI:** 10.1192/j.eurpsy.2025.337

**Published:** 2025-08-26

**Authors:** Y. Xiao, Y. Meng, T. T. Brown, A. C. Tsai, L. Snowden, J. C.-C. Chow, J. Pathak, J. J. Mann

**Affiliations:** 1 Weill Cornell Medicine, Cornell University, New York; 2 UC Berkeley, Berkeley; 3 Harvard Medical School, Boston; 4 Columbia University Irving Medical Center, Columbia University, New York, United States

## Abstract

**Introduction:**

Social determinants of health (SDOH) have been linked to disparities in suicide rates across various demographics, including racial/ethnic groups, sex, age, and geography in the U.S. However, most studies have focused on individual or selected SDOH, rather than examining comprehensive, multi-dimensional SDOH factors. A more nuanced understanding of how clusters of SDOH contribute to suicide disparities across counties is needed to inform targeted prevention strategies.

**Objectives:**

To identify multi-dimensional SDOH county clusters and estimate their geographic and temporal associations with county-level suicide rates.

**Methods:**

This study used national SDOH data from 3,109 U.S. counties over three time periods (2009, 2014, and 2019), matching them with county-level suicide rates from the National Vital Statistics System aggregated into three-year periods (2008-2010, 2013-2015, and 2018-2020). A total of 284 county-level SDOH variables, spanning six domains (social context, economic context, education, physical infrastructure, healthcare context, and natural environment), were analyzed using unsupervised machine learning algorithms to identify SDOH clusters. Associations between SDOH clusters and county-level suicide rates were estimated using negative binomial and LASSO regression.

**Results:**

Three distinct SDOH clusters were identified (Figure 1):

- Cluster 1 (“REMOTE”) included rural counties with elderly, marginalized populations and substandard housing.
- Cluster 2 (“COPE”) represented counties with complex family dynamics, overburdened health systems, poverty, and extreme heat challenges.
- Cluster 3 (“DIVERSE”) encompassed densely populated areas with immigrants, racial/ethnic minorities, environmental challenges, and economic inequality.

Geographically, REMOTE was more common in North and Central U.S., COPE in the South and Central U.S., and DIVERSE along the coasts. Suicide rates were highest in REMOTE counties, especially among men. COPE counties had elevated suicide rates among Whites, while DIVERSE counties saw higher rates among women and Black/Hispanic populations. Most counties (70%) remained within the same cluster over time, with stable suicide rate associations.

**Conclusions:**

This study identified three multi-dimensional SDOH clusters that were associated with varying suicide rates across U.S. counties. These clusters offer insight into the social and environmental conditions contributing to suicide risk. Future prevention strategies should focus on addressing the distinct challenges within each cluster, such as housing inadequacies, healthcare access, and economic inequality, to reduce overall suicide rates and related disparities.

**Disclosure of Interest:**

None Declared

